# Supplementary figures and images for: RNA-Spray-Mediated Silencing of Fusarium graminearum AGO and DCL Genes Improve Barley Disease Resistance
Source: Front Plant Sci. 2020 Apr 29;11:476. doi: 10.3389/fpls.2020.00476 (PMC7202221; doi:10.3389/fpls.2020.00476)

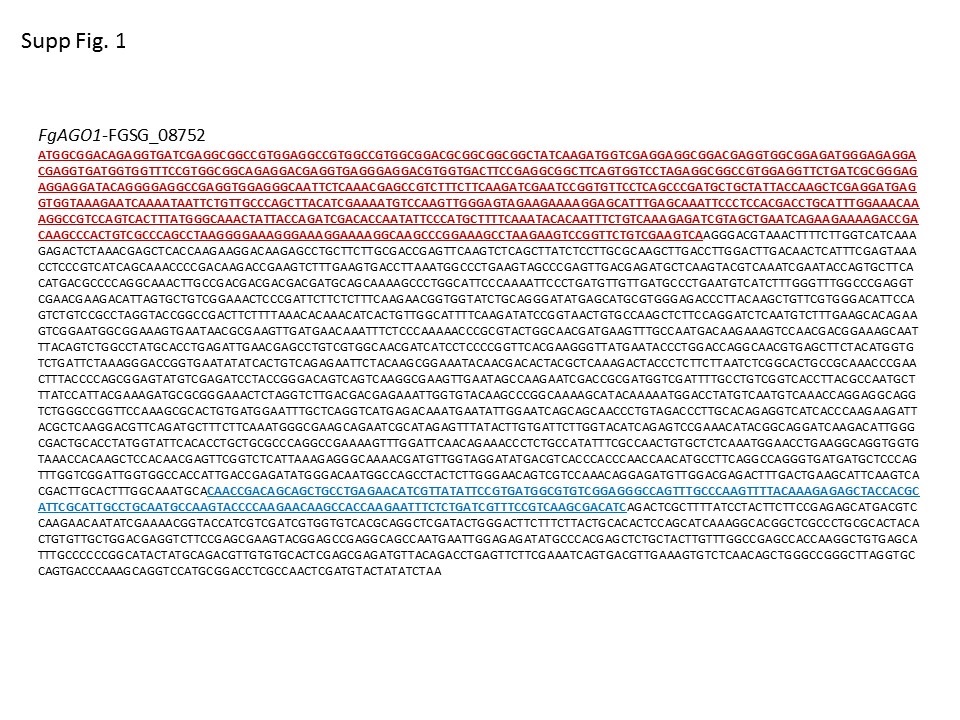

Supplement: FIGURES S1–S4 — Coding sequences (CDS) of the respective Fg target gene with the sequences of the dsRNA marked (blue, tool-designed; red, manually designed). [file Image_1.JPEG]

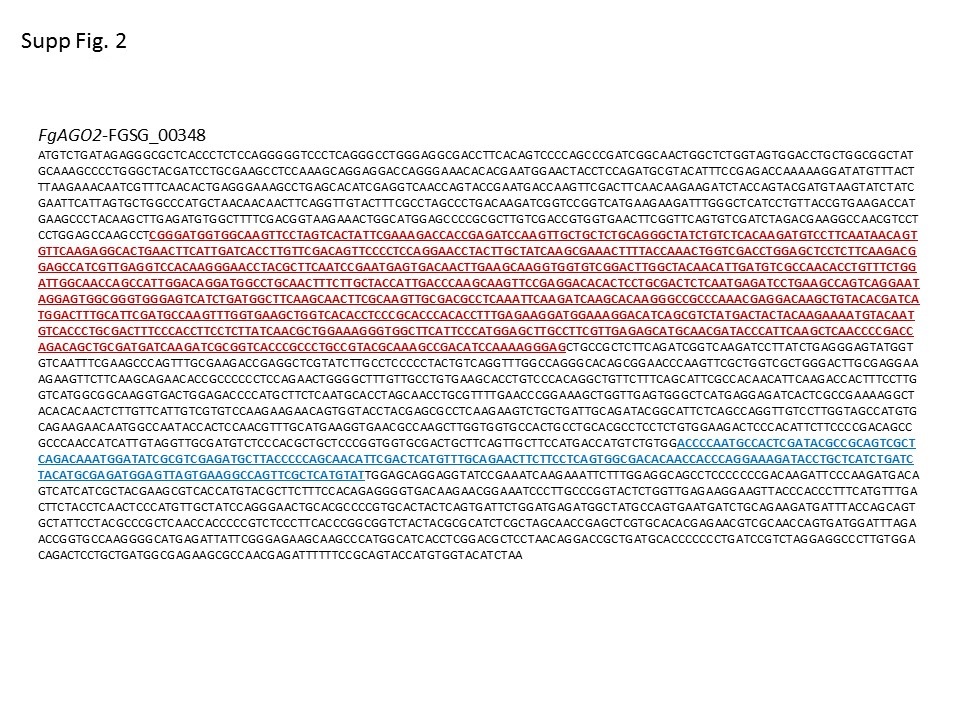

Supplement: Supplementary file 2 [file Image_2.JPEG]

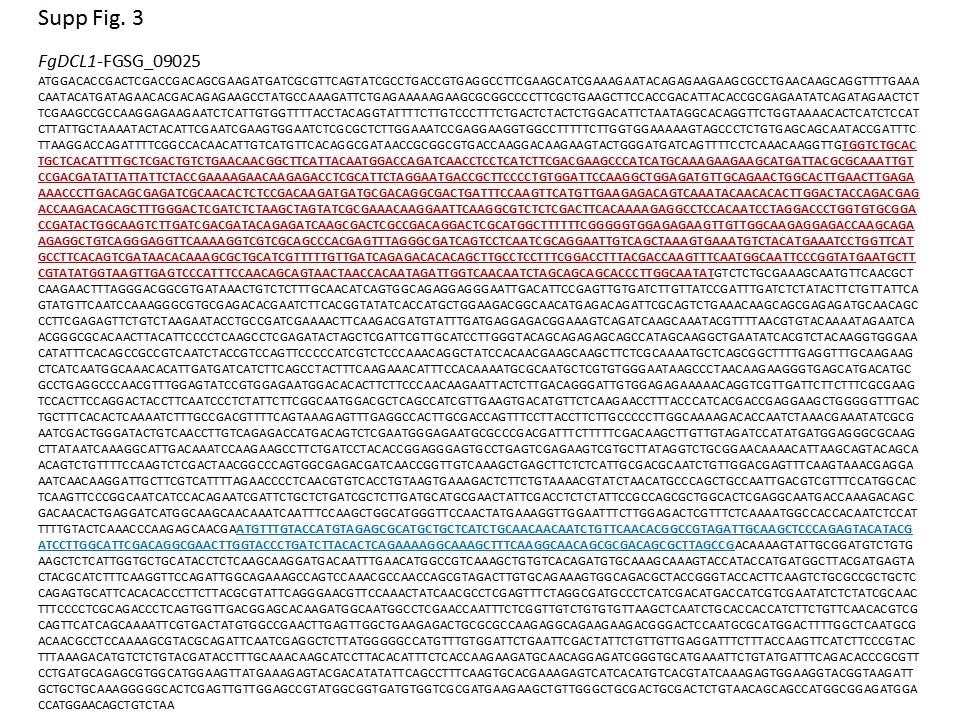

Supplement: Supplementary file 3 [file Image_3.JPEG]

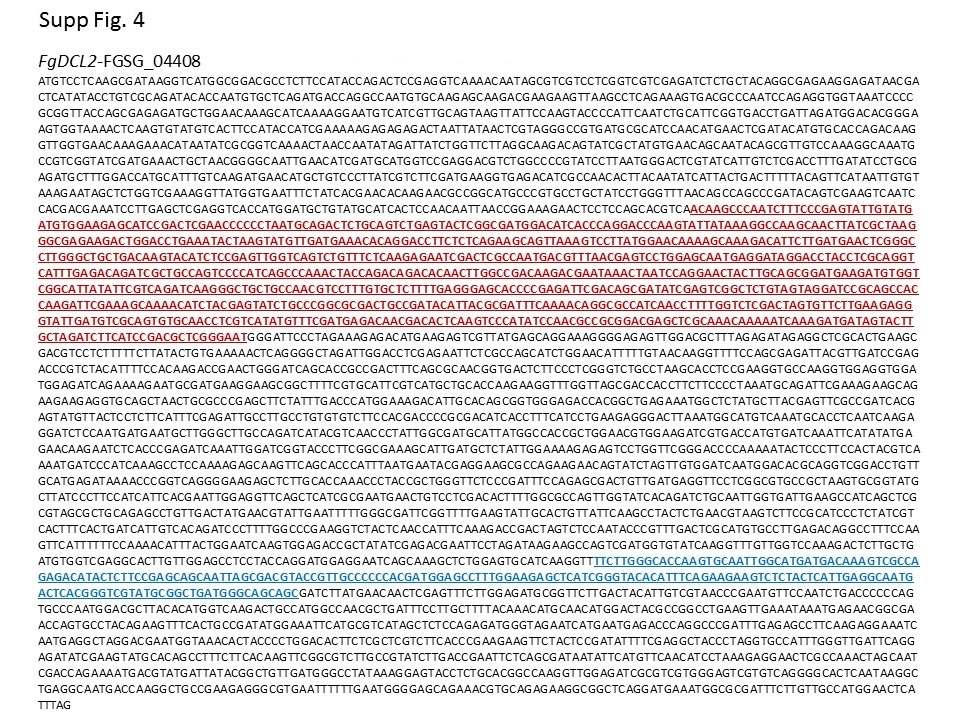

Supplement: Supplementary file 4 [file Image_4.JPEG]

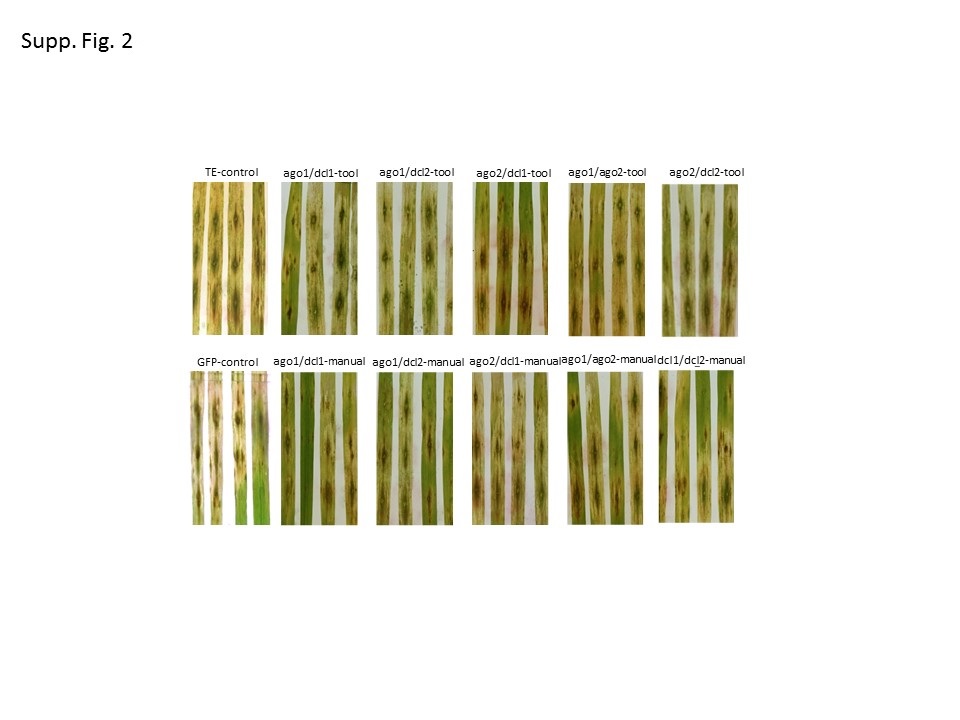

Supplement: FIGURE S5 — Representative pictures of barley (golden promise) leaves sprayed with 10 μg (20 ng/μl) of respective dsRNA in TE-Buffer and the control without dsRNA. DsRNA was applied on the upper half of 10 leaves and 2 days after spraying the leaves were inoculated with three 20 μl droplets of Fg (50,000 spores/ml). The pictures were taken 5 dpi. [file Image_5.JPEG]
