# Supplementary material for: RNA-Spray-Mediated Silencing of Fusarium graminearum AGO and DCL Genes Improve Barley Disease Resistance
Source: Front Plant Sci. 2020 Apr 29;11:476. doi: 10.3389/fpls.2020.00476 (PMC7202221; doi:10.3389/fpls.2020.00476)
Supplement: TABLE S1 — Primers used in this study. [file Table_1.DOCX]

| **Name** | **Sequence** |
| --- | --- |
| FgAGO1_F | TCCTCTCCAATTTCTTCCCCG |
| FgAGO1_R | TGACTTCGACAGAACCGGAC |
| FgAGO2_F | GGGATGGTGGCAAGTTCCTA |
| FgAGO2_R | CTCCCTTTTGGATGTCGGCT |
| FgDCL1_F | TGGTCTGCACTGCTCACATT |
| FgDCL1_R | ATATTGCCAAGGGTGCTGCT |
| FgDCL2_F | ACAAGCCCAATCTTTCCCGA |
| FgDCL2_R | ATTCCCGAGCGTCGGATGA |
| FgAGO1+NotI_F | CTGCGGCCGCTCCTCTCCAATTTCTTCCCCG |
| FgAGO1+NdeI _RC | CTCATATGTGACTTCGACAGAACCGGAC |
| FgAGO2+NotI _F | CTGCGGCCGCGGGATGGTGGCAAGTTCCTA |
| FgAGO2+NdeI_R | CCTCATATGCTCCCTTTTGGATGTCGGCT |
| FgDCL1+NotI _F | CTGCGGCCGCTGGTCTGCACTGCTCACATT |
| FgDCL1+NedI _R | CCTCATATGATATTGCCAAGGGTGCTGCT |
| FgDCL2+NotI _F | CTGCGGCCGCACAAGCCCAATCTTTCCCGA |
| FgDCL2+SalI _RC | CTGTCGACATTCCCGAGCGTCGGATGA |
| FgDCL2+NotI _F | CTGCGGCCGCACAAGCCCAATCTTTCCCGA |
| FgDCL2+BstxI _R | CCAGAGAGGTGGATTCCCGAGCGTCGGATGA |
| Fg.AGO1(PGEMT^*^)F | CAACCGACAGCAGCTGCCTG |
| Fg.AGO1(PGEMT*)R | GATGTCGCTTGACGGAAACG |
| Fg.AGO2 (PGEMT*)F | ACCCCAATGCCACTCGATAC |
| Fg.AGO2 (PGEMT*)R | ATACATGAGCGAACTGGCCT |
| Fg.DCL1 (PGEMT*)F | ATGTTTGTACCATGTAGAGC |
| Fg.DCL1 (PGEMT*)R | CGGCTAAGCGCTGTCGCGCT |
| Fg.DCL2 (PGEMT*)F | TTCTTGGGCACCAAGTGCAA |
| Fg.DCL2 (PGEMT*)R | GCTGCTGCCCATCAGCCGCA |
| Fg.AGO1^*^F NotI | GTGCGGCCGCCAACCGACAGCAGCTGCCTG |
| Fg.AGO1*R SalI | CCGGTCGACGATGTCGCTTGACGGAAACG |
| Fg.AGO2 *F NotI | GTGCGGCCGCACCCCAATGCCACTCGATAC |
| Fg.AGO2*R SalI | CCGGTCGACATACATGAGCGAACTGGCCT |
| Fg.DCL1*F NotI | GTGCGGCCGCATGTTTGTACCATGTAGAGC |
| Fg.DCL1*R SalI | CCGGTCGACCGGCTAAGCGCTGTCGCGCT |
| Fg.DCL2*F NotI | GTGCGGCCGCTTCTTGGGCACCAAGTGCAA |
| Fg.DCL2*R SalI | CCGGTCGACGCTGCTGCCCATCAGCCGCA |
| FgAGO1_F+T7 | TAATACGACTCACTATAGGGTCCTCTCCAATTTCTTCCCCG |
| FgAGO1_R+T7 | TAATACGACTCACTATAGGGTGACTTCGACAGAACCGGAC |
| FgAGO2_F+T7 | TAATACGACTCACTATAGGGGGGATGGTGGCAAGTTCCTA |
| FgAGO2_R+T7 | TAATACGACTCACTATAGGGCTCCCTTTTGGATGTCGGCT |
| FgDCL1_F+T7 | TAATACGACTCACTATAGGGTGGTCTGCACTGCTCACATT |
| FgDCL1_R+T7 | TAATACGACTCACTATAGGGATATTGCCAAGGGTGCTGCT |
| FgDCL2_F+T7 | TAATACGACTCACTATAGGGACAAGCCCAATCTTTCCCGA |
| FgDCL2_R+T7 | TAATACGACTCACTATAGGGATTCCCGAGCGTCGGATGA |
| Fg.AGO1*+T7F | TAATACGACTCACTATAGGGCAACCGACAGCAGCTGCCTG |
| Fg.AGO1*+T7R | TAATACGACTCACTATAGGGGATGTCGCTTGACGGAAACG |
| Fg.AGO2 *+T7F | TAATACGACTCACTATAGGGACCCCAATGCCACTCGATAC |
| Fg.AGO2*+T7R | TAATACGACTCACTATAGGGATACATGAGCGAACTGGCCT |
| Fg.DCL1*+T7F | TAATACGACTCACTATAGGGATGTTTGTACCATGTAGAGC |
| Fg.DCL1*+T7R | TAATACGACTCACTATAGGGCGGCTAAGCGCTGTCGCGCT |
| Fg.DCL2*+T7F | TAATACGACTCACTATAGGGTTCTTGGGCACCAAGTGCAA |
| Fg.DCL2*+T7R | TAATACGACTCACTATAGGGGCTGCTGCCCATCAGCCGCA |
